# The structure of the rat vitamin B_12_ transporter TC and its complex with glutathionylcobalamin

**DOI:** 10.1016/j.jbc.2024.107289

**Published:** 2024-04-16

**Authors:** Marcel Bokhove, Takashi Kawamura, Hideo Okumura, Sawako Goto, Yoshiaki Kawano, Stefan Werner, Franziska Jarczowski, Victor Klimyuk, Akihiko Saito, Takashi Kumasaka

**Affiliations:** 1 Structural Biology Division, Japan Synchrotron Radiation Research Institute (JASRI), Sayo, Hyogo, Japan; 2 Department of Applied Molecular Medicine, Kidney Research Center, Niigata University Graduate School of Medical and Dental Sciences, Chuo-ku, Niigata, Japan; 3 Advanced Photon Technology Division, RIKEN SPring-8 Center, Sayo, Hyogo, Japan; 4 Icon Genetics GmbH, Halle (Saale), Germany

**Keywords:** vitamin B_12_, cobalamin, glutathione, complex, transcobalamin, vitamin transport

## Abstract

Vitamin B_12_ (cobalamin or Cbl) functions as a cofactor in two important enzymatic processes in human cells, and life is not sustainable without it. B_12_ is obtained from food and travels from the stomach, through the intestine, and into the bloodstream by three B_12_-transporting proteins: salivary haptocorrin (HC), gastric intrinsic factor, and transcobalamin (TC), which all bind B_12_ with high affinity and require proteolytic degradation to liberate Cbl. After intracellular delivery of dietary B_12_, Cbl in the aquo/hydroxocobalamin form can coordinate various nucleophiles, for example, GSH, giving rise to glutathionylcobalamin (GSCbl), a naturally occurring form of vitamin B_12_. Currently, there is no data showing whether GSCbl is recognized and transported in the human body. Our crystallographic data shows for the first time the complex between a vitamin B_12_ transporter and GSCbl, which compared to aquo/hydroxocobalamin, binds TC equally well. Furthermore, sequence analysis and structural comparisons show that TC recognizes and transports GSCbl and that the residues involved are conserved among TCs from different organisms. Interestingly, haptocorrin and intrinsic factor are not structurally tailored to bind GSCbl. This study provides new insights into the interactions between TC and Cbl.

Vitamin B_12_ (cobalamin [Cbl]) is a cofactor in two essential enzymatic processes in the human body: (i) methionine synthesis and (ii) the conversion of methylmalonic acid to succinyl-CoA, which appears during the catabolism of branched-chain amino acids and odd-chain fatty acids (1, 2). Vitamin B_12_ is produced only by certain microorganisms, while all higher organisms obtain B_12_ from gastro-intestinal microbiota (in the case of herbivores), from animal-derived food, or from their environment (in the case of carnivores and omnivores) (3). In humans, B_12_ is mainly obtained from the diet, that is, meat, fish, dairy products, or supplements. B_12_ uptake is a finely orchestrated process involving a number of structurally related high-affinity B_12_-binding proteins, which protect B_12_ and carry it through cell membranes *via* specific protein–receptor interactions. The proteins in question are salivary haptocorrin (HC), gastric intrinsic factor (IF), and transcobalamin (TC), the latter circulating in blood together with internally produced HC (4, 5, 6, 7, 8, 9). HC, IF, and TC can only release B_12_ after proteolytic degradation (7). B_12_ deficiency is associated with various diseases, including neurological disorders and anemia (7, 10). Even though vitamin B_12_ is utilized in only two processes, it is an essential molecule and the life of animal kingdom is not possible without it.

The reactive center of Cbl is formed by cobalt (Co), coordinated by four equatorial corrin-ring nitrogens (11). The isolated Cbl at neutral pH and ambient oxygen is kept in an inert cob(III) alamin “base on” state (Co in +3 oxidation state), where the fifth lower (α) axial site is represented by the nitrogen of the dimethylbenzimidazole base containing side chain of Cbl. The sixth upper (β) axial site usually contains water, cyanide, a variety of nucleophiles, as well as histidine in the case of TC (11, 12). As a coenzyme bound to a respective site, Cbl exists in the reactive “base off” state and has a β-axial methyl or adenosyl group (1, 2). Hydroxocobalamin (HOCbl) can coordinate thiolates such as GSH, which has been described as early as 1964 (13). Given the high intracellular concentration of GSH (0.5–10 mM), it has been hypothesized that glutathionylcobalamin (GSCbl) is the natural form (14, 15) acting as a processing intermediate. Indeed, GSCbl has been observed *in vitro* as an intermediate in human and *Caenorhabditis elegans* methylmalonic aciduria and homocystinuria type C protein, also known as MMACHC or CblC, an enzyme in the Cbl-processing pathway (16, 17). A detectable fraction of GSCbl has been purified from living cells (18, 19). GSCbl binds with a high affinity to the Cbl exporter MRP1 (9), which translocates Cbls to the bloodstream, where they will circulate and bind to TC (though the TC–GSCbl complex has not yet been described in blood plasma). Several papers demonstrate that GSCbl is more reactive than a number of other Cbl forms, when generating methylcobalamin or adenosylcobalamin *in vitro* (14, 20). The crystal structure of GSCbl has been elucidated (21); however, there is no data whether Cbl transporters recognize and form a complex with GSCbl.

In this paper, we present the complex crystal structure between rat transcobalamin (rTC) and GSCbl. We show how TC recognizes GSCbl and that the interactions with GSH in TC are less rigidly arranged than in other proteins. It has been suggested that GSCbl may be an effective form of B_12_ to treat patients and TC can play its part in the safe delivery of GSCbl to the tissues.

## Results and discussion

### The 1.3 Å resolution structure of rTC and interaction with HOCbl

**Figure 1:**
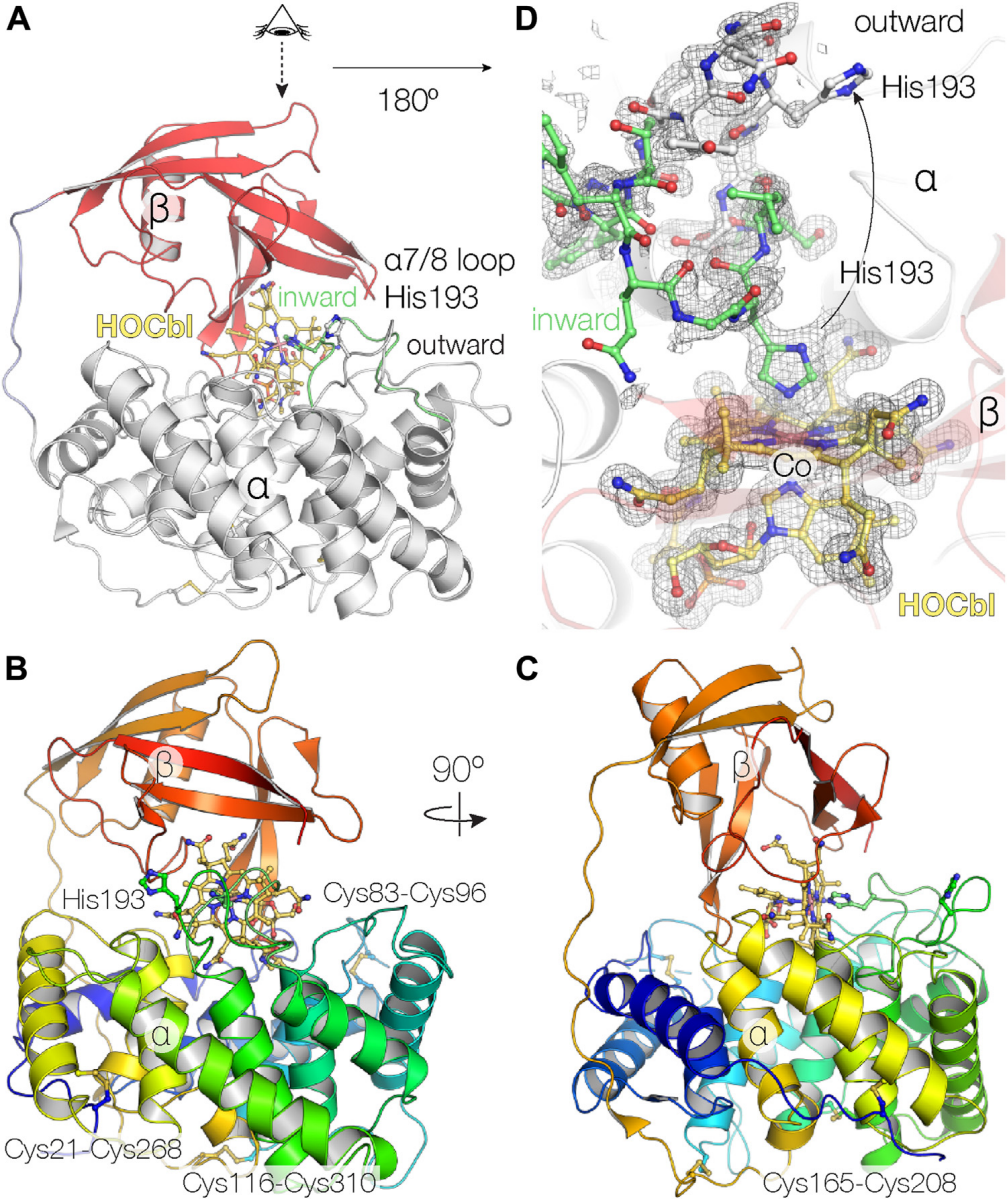
**The rTC–HOCbl complex.***A*, Cbl is bound between the α- and β-domains (*whiteandredcartoons*, respectively), connected by an interdomain linker (*blue*). *B* and *C orthogonal views* on rTC *rainbow-colored blue to red* from N to C terminus with the ligand vitamin B_12_, His193, and the disulfide-bonded cysteines as *ball* and *sticks*. *D*, the His193 loop has two conformations. The electron density omit map is contoured at 2 σ (*gray mesh*). Cbl, cobalamin; HOCbl, hydroxocobalamin; rTC, rat transcobalamin.

For this work, rTC was recombinantly expressed in *Nicotiana benthamiana* tobacco plants. No crystals of apo-rTC could be obtained, but when vitamin B_12_ was added, for example, in the HOCbl form, clusters of bright cherry-colored crystals readily appeared that diffracted to 1.3 Å resolution (Table 1). Like the previously elucidated bovine and human TC-Cbl structures (12), recombinantly expressed rat TC consists of two domains: (i) an N-terminal, disulfide-bonded, α-helical six-hairpin glycosidase-like α-domain and (ii) a C-terminal β-stranded ubiquitin-like β-domain, the two linked by an interdomain loop (Fig. 1, *A*–*C*; Figs. S1 and S2). The α helices in the α-domain are held together by four disulfide bonds Cys21–Cys268, Cys83–Cys96, Cys116–Cys310, and Cys165–Cys208 (Figs. 1, *B* and *C*; S1). This disulfide-bonding pattern is like that found for human TC, while bovine TC has only three disulfide bonds (12) (Fig. S1). The 12 α-helices in the α-domain form a cylindrical structure with a central hole closed off by the 13th α-helix, which is followed by the interdomain linker (Fig. S2*A*). The β-domain consists of a β hairpin (β1′/β2′), an α-helix (α1′), a β hairpin (β3′/β4′), and another β hairpin (β6′/β8′) that forms a β sheet with β1′/β2′. Human and bovine TC share 75.6% and 69.9% sequence identity with rTC, which can be superimposed onto human and bovine TC with a Cα rmsd of 1.25 and 1.20 Å, respectively.

**Table 1:** Data collection and refinement statistics

Parameters	TC HOCbl	TC GSCbl
Data collection		
PDB ID	8IXU	8IXT
Wavelength (Å)	1.0	1.0
Resolution range (Å)	29.88–1.3 (1.346–1.3)	36.24–1.2 (1.243–1.2)
Space group	*P*2_1_2_1_2_1_	*P*2_1_2_1_2_1_
Unit cell (Å, °)	51.31 54.53 142.87 90 90 90	51.10 55.06 143.67 90 90 90
Total reflections	1 322 524 (129 055)	4 259 423 (409 338)
Unique reflections	99 382 (9 805)	127 253 (12 501)
Multiplicity	13.3 (13.2)	33.5 (32.7)
Completeness (%)	99.97 (99.98)	99.90 (99.30)
Mean I/sigma(I)	12.57 (0.75)	21.06 (2.10)
Wilson B-factor (Å^2^)	16.60	13.08
R-merge	0.1082 (3.005)	0.09226 (1.711)
R-meas	0.1125 (3.126)	0.09367 (1.738)
R-pim	0.03077 (0.8533)	0.01602 (0.2999)
CC1/2	1.0 (0.319)	1.0 (0.788)
CC∗	1.0 (0.696)	1.0 (0.939)
Refinement		
Reflections used in refinement	99 379 (9803)	127 240 (12499)
Reflections used for R-free	1 576 (151)	2 016 (200)
R-work	0.1603 (0.3268)	0.1607 (0.2403)
R-free	0.1714 (0.3442)	0.1705 (0.2602)
CC (work)	0.972 (0.662)	0.966 (0.872)
CC (free)	0.971 (0.601)	0.973 (0.829)
Number of nonhydrogen atoms	3 719	3 760
Macromolecules	3 260	3 187
Ligands	193	217
Solvent	355	460
Protein residues	400	401
Model statistics		
RMS (bonds) (Å)	0.006	0.006
RMS (angles) (º)	0.90	0.99
Ramachandran favored (%)	99.75	99.24
Ramachandran allowed (%)	0.25	0.76
Ramachandran outliers (%)	0.00	0.00
Rotamer outliers (%)	0.00	0.00
Clash score	1.78	1.51
Average B-factor (Å^2^)	27.27	19.66
Macromolecules	27.01	18.99
Ligands	16.95	12.82
Solvent	32.74	25.97
Number of TLS groups	4	5

Statistics for the highest resolution shell are shown in parentheses.

GSCbl, glutathionylcobalamin; HOCbl, hydroxocobalamin; TC, transcobalamin; TLS, translation, libration, and screw-rotation.

Superimposing the α-domains results in a good agreement between α-domains and B_12_ molecules, discussed below, but the β-domain of rTC shows a different orientation with deviations up to 3.5 Å, suggesting high adaptability of rTC (Fig. S2*B*) without limiting the interaction with Cbl. The ligand anchors β3′/β4′/β5′/β6′/β7′ and the loop connecting β7′-β8′ of the β-domain; however, β1′-β2′-α1′ deviate significantly mainly due to elongated residues Arg345, Arg361, and Arg363, which are pushing these secondary structure elements away from the rest of the β-domain compared to the bovine and human proteins (Fig. S2*B*). The way the α-and β-domains accommodate Cbl is in agreement with the observation that association of α/β-domain in Cbl transporters is Cbl-dependent and that isolated α- and β-domains have the ability to bind Cbl (22, 23, 24). For example, there are organisms that have only the β-domain as B_12_ transport proteins (25).

In the shown structure, Cbl is sandwiched between the α- and β-domains of rTC in a base-on conformation (Fig. 1, *A* and *D*). This crystal, obtained at pH 7.5, indicates that the α7/α8 loop (that interacts with the Co site of HOCbl) shows a simultaneous presence of inward- and outward-facing conformations (Fig. 1*D*). Both conformations show high mobility, but there is a strong continuous electron density from the Co ion to His193 Nε2 (Co–N distance of 2.1 Å) (Fig. 1*D*), which has been observed previously for bovine TC, as well as in human TC, though to a lesser extent (12). This histidine residue is conserved in TC, but mostly absent in IF and HC, although porcine HC does have the histidine (Fig. S1). When the β-axial site is occupied, for example, in the case of cyanocobalamin, TC only shows the outward facing conformation (12, 26). The presence of both α7/α8 loop conformations in one structure indicates how TC can adapt to different substrates.

### TC has the ability to bind GSCbl

**Figure 2:**
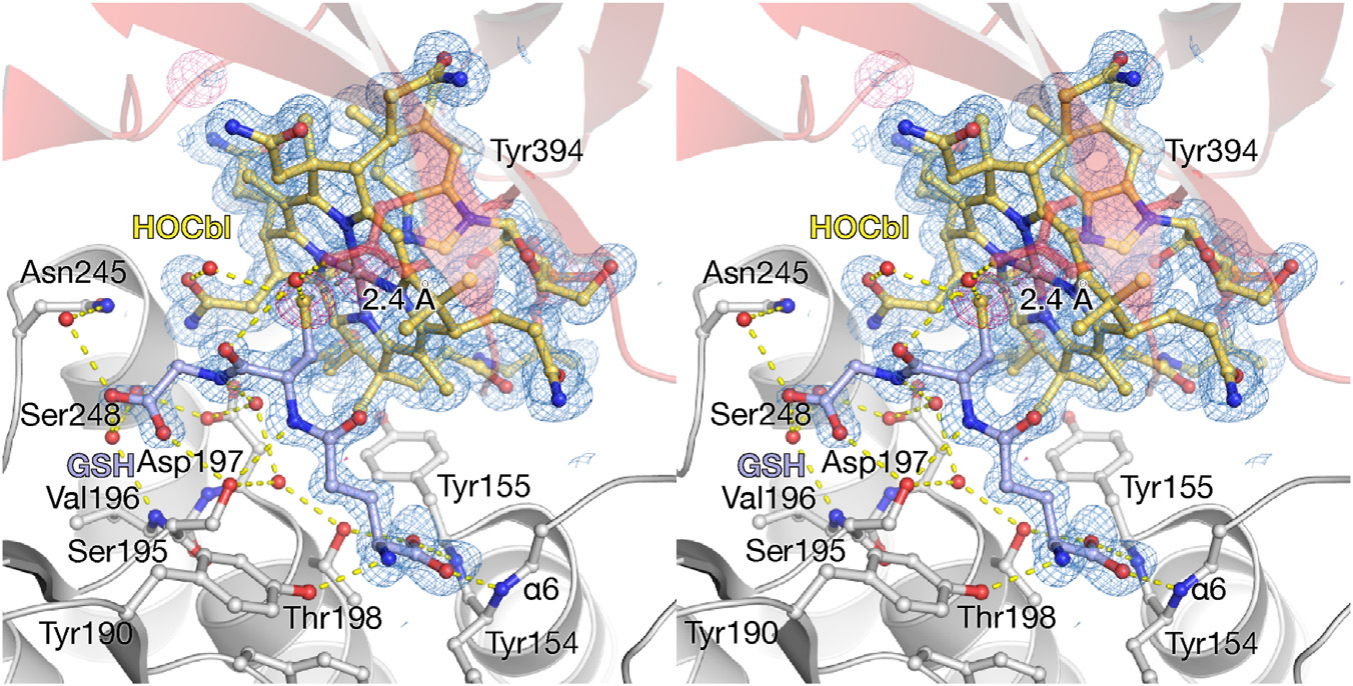
**Stereo figure of the rTC–GSCbl complex.** Cbl is *yellow* and GSH is *blue*. The α- and β-domains are shown as *white and red cartoons*, respectively. Residues that interact with GSH are labeled and shown as *sticks*. Hydrogen bonds between GSH, residues, and solvent are indicated by *yellow dashes*. The 2mFo-DFc electron density (*blue mesh*) is contoured at 1 σ; the anomalous difference map collected at λ = 1.7 Å is contoured at 4 σ (*red mesh*). Cbl, cobalamin; GSCbl, glutathionylcobalamin; rTC, rat transcobalamin.

**Figure 3:**
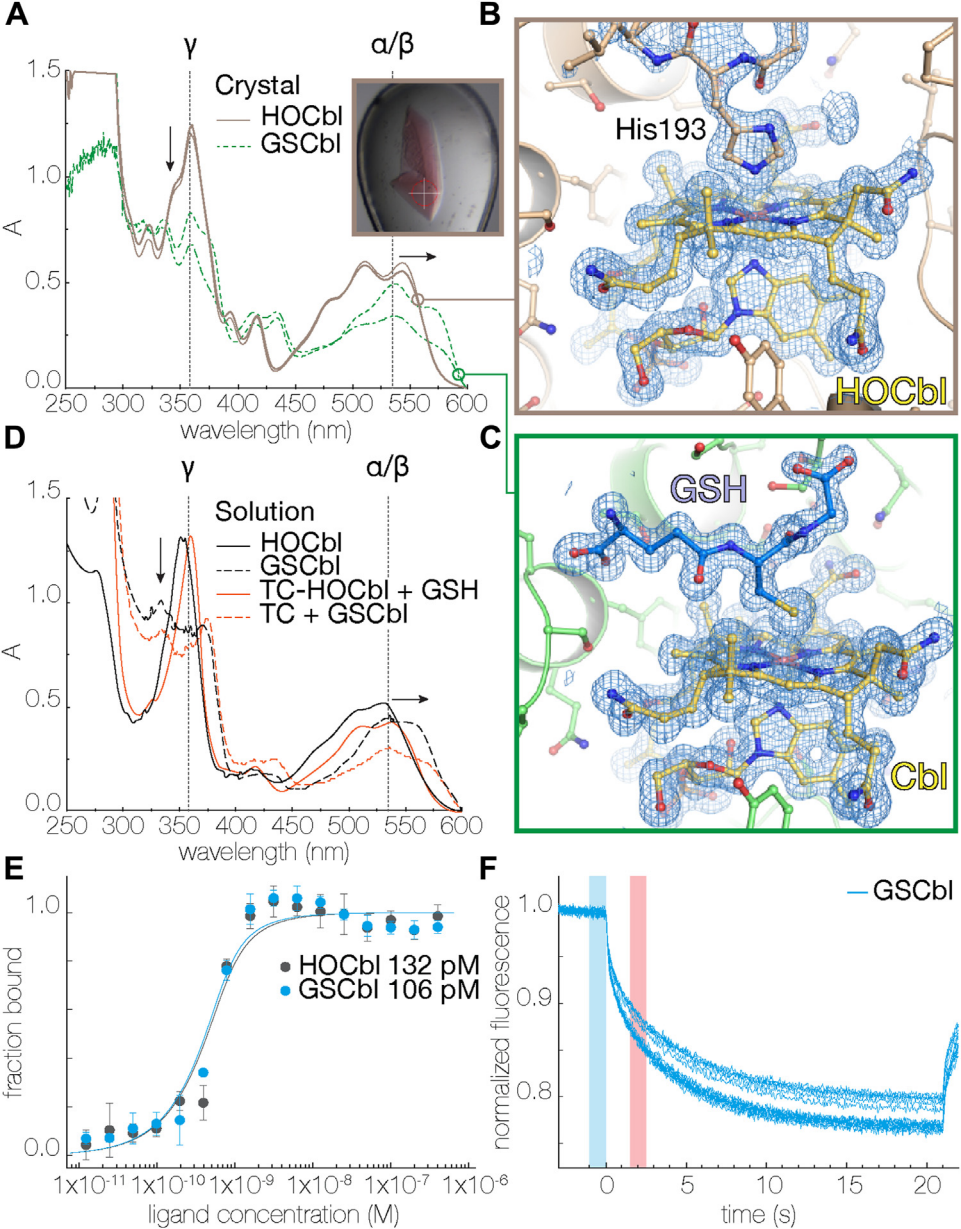
**TC binds GSCbl.***A* single-crystal microspectrophotometry spectra at 100 K. rTC crystals (two conditions) in complex with HOCbl (*brown*) or GSCbl (*green*), inset shows an rTC-GSCbl crystal (*white cross* is 100 μm). *B*, rTC-HOCbl structure with the β-axial ligand His193. *C* rTC-GSCbl structure with the β-axial ligand GSH. 2mFo-DFc electron density maps are contoured at 1 σ (*blue mesh*). *D*, free and protein-bound spectra of HOCbl and GSCbl. In *A* and *D,* α/β and γ are typical HOCbl absorbance spectra peaks. *Arrows* indicate spectral changes. *E*, microscale thermophoresis binding curves of rTC with HOCbl and GSCbl. Error bars are SDs of n = 5 measurements. *F*, fluorescence thermophoresis curves. GSCbl, glutathionylcobalamin; HOCbl, hydroxocobalamin; rTC, rat transcobalamin; TC, transcobalamin.

We obtained crystals of rTC that diffracted to 1.2 Å resolution (Table 1) at pH 5.5 in the presence of GSH and to our surprise we observed continuous electron density emanating from the Co of the Cbl ring system corresponding to GSH (Fig. 2). This is likely due to the dissociation of His193 in its protonated state, resulting in the outward-facing conformation previously observed at pH 7.5 (Fig. 1*D*). Subsequently, crystals of the rTC–GSCbl complex could be prepared seamlessly, irrespective of pH, by mixing GSH and HOCbl prior to addition to the protein, since GSCbl formation is very tight (15). To confirm the presence of GSCbl in the rTC crystals, we performed microcrystal spectrophotometry experiments on rTC cocrystallized with either HOCbl or GSCbl and compared the resulting spectra (Fig. 3). rTC-HOCbl crystals showed a base-on His-Cbl spectrum (Fig. 3*A*) (11, 27, 28), which is comparable to HOCbl in a solution containing histidine or HOCbl bound to TCs from other host organisms (Fig. 3*D*). rTC-GSCbl crystals show the typical spectrum of GSCbl (Fig. 3*A*) when compared to solutions of GSCbl or TC-GSCbl (Fig. 3*D*), that is, the collapse of the γ peak and the α/β red shift (11, 13, 27, 28). After collecting the spectra, the same crystals were used to obtain diffraction data, clearly showing the binding of His193 in the α7/α8 loop to HOCbl in rTC-HOCbl (Fig. 3*B*) and the presence of the GSH-bound Cbl in rTC-GSCbl (Fig. 3*C*). Furthermore, microscale thermophoresis (MST) analysis shows that both HOCbl and GSCbl bind with a similar affinity to rTC under these experimental conditions (Fig. 3, *E* and *F*).

GSH binds at the β-axial site, located between Cbl and the α-domain of rTC. Therefore, most interactions are formed with residues located in the α-domain (Fig. 2). The Co–S bond is approximately 2.4 Å, the Co–dimethylbenzimidazole base bond is 2.2 Å (Fig. 2) and the corrin fold angle is 6.6°, which is defined as the butterfly angle between N21, C4, C5, C6, N22 C9, C10, and C10, C11, N23, C14, C15, C16, and N24. In our structures, the distances and angles are reliable since the resolution of the maps is among the highest for vitamin B_12_ in the protein databank (29). The corrin fold angle is similar to that of HOCbl bound to TC (5.3°), where both the α- and β-axial site bond lengths are 2.1 Å. Currently there is only one other GSCbl structure available (21); however it is difficult to compare GSCbl in the protein-bound state with the small-molecule crystal structure since they are structurally quite different, suggesting that crystal packing interactions can affect the corrin fold angle. Similar discrepancies are observed for the protein-bound and small-molecule crystal structures of HOCbl.

The majority of the 14 polar interactions between GSH and rTC are formed through solvent molecules (Fig. 2). However, direct side chain interactions are present between Ser195, Thr198, and GSH. The strongest interaction is found between the α-helix dipole of α6 (Tyr154 and Tyr155), which interacts with the carboxylate of the γ-glutamate group of GSH. A detailed scheme indicates the interactions between TC and GSCbl and the conservation of the interacting residues, see Fig. S3. Most of the residues that interact with GSH are conserved among TC sequences (Figs. S1 and S3).

### TC stabilizes GSCbl

The interaction between the α-helix dipole of TC and GSH (Fig. 2) is found in many GSH-binding proteins, including CblC (30). Interestingly, CblC, which binds base-off Cbl, is an important B_12_-processing enzyme that converts, for example, methylcobalamin, adenosylcobalamin, cyanocobalamin to activated cob(II) alamin using a nucleophilic displacement system (31, 32). In the enzymatic reaction performed by CblC, GSH acts as an acceptor for the departing group of the Cbl substrate. The crystal structure of CblC in complex with a B_12_ antivitamin and GSH shows that CblC has substrate-binding residues to interact with GSH, which are mutated in patients suffering from B_12_ deficiency diseases (11, 30). No clinical point mutations (uniprot.org; human TC UniProtKB entry P20062) are found yet for residues in TC that interact with GSH; however, neither are there for those interacting with Cbl. It has been suggested that CblC is capable of protecting Cbl from reduced GSH or removing GSH altogether (20, 33). The crystal structure shows that residues in CblC are optimally oriented to interact with GSH such that the thiol group is located 6.2 Å from the Co atom, thereby removing GSH from Cbl as suggested by spectroscopic studies (20, 32, 33). In solution, the spectrum of free GSCbl slowly changes to the spectrum resembling HOCbl under aerobic conditions *in vitro*, possibly accelerated by light and photodecomposition, even though the samples were stored in the dark (Fig. S4: *red spectra* 1–4). This happens because GSH-initiated reduction of Cbl causes the spontaneous formation of radicals and oxidation products (34). TC-bound GSCbl remains stable for a prolonged period of time (Fig. S4: *blue spectra* 1–4), suggesting that TC can sequester and stabilize GSCbl. A similar observation has been made for oligonucleotide-substituted Cbls, which quickly undergo photolysis, but not when they are bound to TC (35). Once TC has been degraded in the lysosome, the bound Cbl form is liberated and processed by CblC for use as coenzyme.

### Steric hindrance might prevent GSCbl from binding to HC and IF but not TC

**Figure 4:**
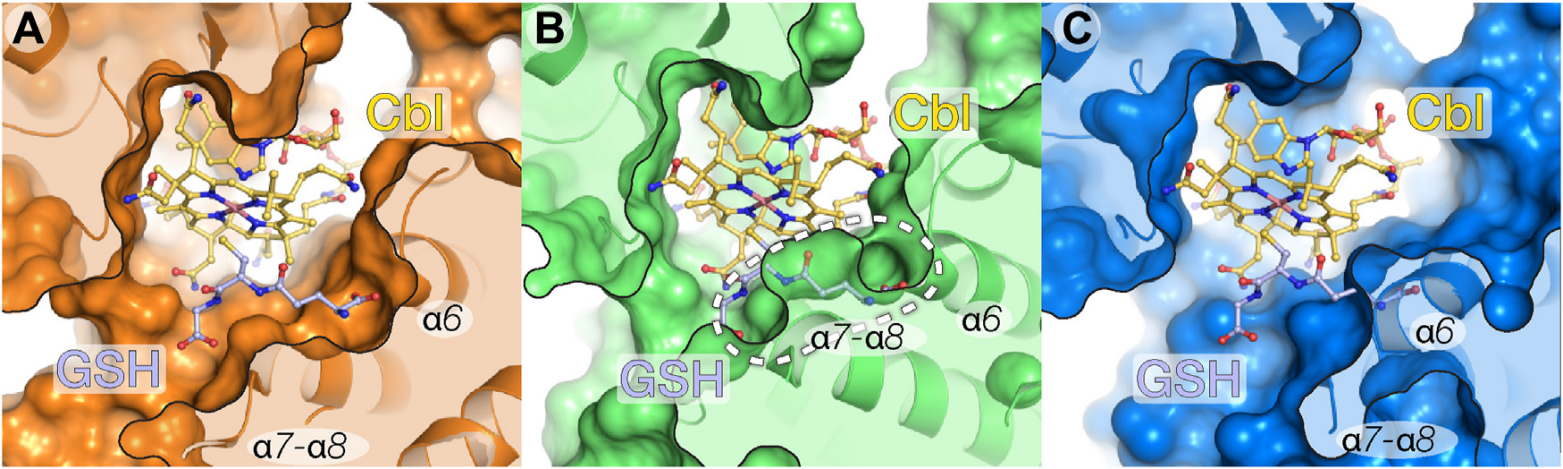
**The substrate-binding pocket of B**_**12**_**transporters.***A* rat transcobalamin. *B*, human haptocorrin (PDB ID 4KKI) (37). *C*, human intrinsic factor (PDB ID 2PMV) (36). B_12_ and GSH are indicated by *yellow and blue sticks*, respectively. In haptocorrin and intrinsic factor, GSH is modeled by superimposing GSCbl onto the bound cobalamin. GSCbl, glutathionylcobalamin.

Remarkably, only inside the binding pocket of TC there is sufficient space for GSH to bind (Fig. 4*A*), while analysis of GSCbl superimposed onto the bound CNCbl shows that steric clashes might occur in HC and IF (PDB IDs 4KKI and 2PMV, respectively) (36, 37) (Fig. 4, *B* and *C*). The same analysis shows that the substrate-binding pockets of human and bovine TC (PDB IDs 7QBF and 2V3N, respectively) (26, 38) like those in rat TC provide space for GSCbl (Fig. S5, *A* and *B*). The histidine (that forms a complex with the Co atom of HOCbl in TCs) is located in the α7/α8 loop. This histidine is absent in IF and HC of most species and although the α7/α8 loop is present in HC, it is shorter and moved slightly aside from the β-face of Cbl in IF (Figs. S1 and S6). When the β site of Cbl is occupied by a strong ligand such as cyanide or GSH, the TC α7/α8 loop is disordered (bovine TC) or folds outward into the solvent (rat and human TC) and forms little hydrogen bonds with the rest of the protein. However, in HC, the α7/α8 loop is sandwiched between the α-domain and the C terminus of the β-domain and forms hydrogen bonds, thereby the α7/α8 loop occupies the location of the GSH-binding site compared to the GSCbl-bound form of rat TC (Figs. 4*B* and S6). Although the α7/α8 loop is shortened in IF, the α6 helix might prevent the GSH from binding because the helix is extended by one turn occupying the GSH-binding site (Figs. 4*C* and S6). Furthermore, the γ-glutamate carboxylate group of GSH cannot form hydrogen bonds with the α-helix dipole of α6. These analyses indicate that HC and IF might have a reduced ability to efficiently recognize and bind GSCbl. More conclusive details on the mechanism of GSCbl recognition by IF and HC require additional experimental studies.

### Adaptability of the TC α-domain affects binding of β axially substituted Cbls

Comparison of the different Cbl transporter complexes can provide a more general explanation for the variation in substrate recognition. Proteins interfaces, surfaces and assemblies analysis (https://www.ebi.ac.uk/pdbe/pisa/) (39) of the rat TC–Cbl complex structures suggests that the favorability of interactions ranked from high to low is Cbl/β domain >Cbl/α domain >α/β-domain. The buried surface area between HOCbl/GSCbl and the β domain of rTC is approximately 560 Å^2^, while buried surface area with the α-domain is approximately 560 and 430 Å^2^ for HOCbl and GSCbl, respectively. The surface area between α- and β-domain is 530 and 440 Å^2^ for HOCbl and GSCbl, respectively. A similar pattern is observed for the other Cbl transporters. The main difference among the latter two collections of interfaces is due to His193 and the α7/8 loop in the α-domain, which interacts with both the Co^3+^ and the β-domain in the HOCbl-bound structure. The difference in buried surface area with the α-domain and the α/β-domain interface between HOCbl and GSCbl is however compensated by GSH.

Even though there is a substantial buried surface area between the α- and β-domain, Cbl is required for α- and β-domain association in Cbl transporters (22, 23). Cbl binds sequentially to the β-domain of Cbl transporters and then the α-domain closes onto the β-domain–Cbl complex (22, 23, 24, 35), which is consistent with PISA analysis. Previously it has been shown that the vitamin B_12_ transporter TC is highly tolerant toward Cbl analogs that carry larger substitutions at the β-axial site including, for example, GSH (Figure 2, Figure 3, Figure 4) and 18-, 25-, or 39-nt long oligonucleotides (35, 40). Interestingly, HC and IF are much less amenable to bulky substitutions (Figs. 4 and S6), even though all three are evolutionary related (41). Different β axially substituted Cbls can bind to the β-domains of the Cbl transporters, because there are only few interactions between the Cbl β-face and the β-domain (Figure 1, Figure 2, and S3). However, when there are secondary structure elements in the α-domain, such as the α7/8 loop and α6, which are unable to accommodate bulky features, these ligands could quickly dissociate (35). In other words, the β-domain performs the initial binding while the α-domain, or the interplay between α- and β-domain, in the case of HC for example (37), performs the selection. Consequently, the α–Cbl–β sandwich complex between HC/IF and Cbl might be energetically less favorable and is not always properly formed, if Cbl has some “inconvenient” β-groups, which could explain the high exchange rate of oligonucleotide-substituted Cbls bound to HC or IF (35). Under the aforementioned circumstances, the affinity of Cbl for HC/IF might be decreased in comparison to TC (Figs. 4 and S6) (35).

Our structure of rTC in complex with GSCbl clearly displays the broad specificity of TC concerning β groups of Cbl. In addition, it indicates that the α7/8 loop and α6 are the secondary structure elements that have a remarkable adaptability. This feature allows TC to accept many different substrates that could be of interest for the delivery of specialized β axially substituted Cbls for medical applications.

## Experimental procedures

### Cloning, protein production, and purification

To obtain the apo form of rTC, the protein was expressed in *N. benthamiana* (tobacco) plants (42). The mRNA sequence of rTC was taken from Genbank (AF054810) and cDNA was synthesized at Geneart; synthetic DNA was cloned into a MagnICON tobacco mosaic virus-based expression vector downstream of a rice α-amylase apoplast targeting sequence, followed by a 6xhistidine tag and an enterokinase cleavage site resulting in construct pICK21104 (Icon Genetics, icongenetics.com). *N. benthamiana* plants were infiltrated, and the green parts were harvested after 7 days and frozen at approximately 200 K (42). Frozen plant material was thawed, homogenized, and clarified by centrifugation at 16,000*g* and applied to a nickel IDA column (Resindion). rTC was eluted with 10 mM sodium phosphate, pH 7.5; 150 mM NaCl; 400 mM imidazole, followed by dialysis against PBS pH 7.5. Dialyzed elution fractions were pooled and frozen for shipment.

After shipment the sample was thawed quickly by hand and concentrated to 0.5 ml at 9 mg/ml using an AMICON concentrator device (Thermo Fisher Scientific) with a 10 kDa molecular weight cut-off filter. Centrifuged and concentrated rTC was injected in two rounds onto a 10/300 Superdex 200 increase column connected to an Äkta pure system (Cytiva) pre-equilibrated with 20 mM Hepes pH 8.0; 1 M NaCl. The fractions were analyzed on an SDS-PAGE gel, and the peak fractions were pooled, concentrated, and diafiltrated with 20 mM Hepes pH 8.0; 100 mM NaCl; optionally including a 2:1 M ratio of reduced and oxidized GSH (2 mM GSH and 1 mM GSSG) (Wako) to 8.6 mg/ml and stored at 4 °C.

### Crystallization and structure determination

rTC at 8.6 mg/ml was subjected to crystallization trials using an NT8 crystallization robot (Formulatrix) with the PACT and JCSG-plus ECO screens (Molecular Dimensions) in MRC2 96-well plates (Molecular Dimensions) by mixing 200 nl protein with an equal amount of reservoir solution. No crystals were obtained for the apoprotein; therefore, 8.6 mg/ml rTC was mixed with HOCbl acetate (Tokyo Chemical Industries), also referred to as vitamin B_12_ or HOCbl, in a 1:1.2 M ratio. In the initial crystallization trials, apo-rTC in the GSH-containing buffer was mixed with HOCbl, which resulted in the GSCbl complex structure (8IXT); in the absence of GSH, we could obtain the HOCbl complex structure (8IXU). For subsequent experiments, GSCbl was prepared by simply mixing HOCbl acetate and reduced GSH in a 1:10 M ratio in 20 mM Hepes pH 8.0; 100 mM NaCl, reflecting the initial ratio where GSCbl was found. rTC-GSCbl was prepared by mixing purified rTC with a 1:1.2 ratio of purified GSCbl.

Bright cherry–colored crystals readily appeared within 2 days growing mostly from precipitate in 10 and 20 different conditions for JCSG-plus and PACT, respectively. This publication mainly discusses structures obtained in two different pH conditions: A higher pH crystal in PACT G5: 200 mM sodium nitrate; 100 mM Bis-Tris propane pH 7.5; 20% PEG 3350 (w/v) (8IXU) and a lower pH crystal in JCSG-plus A2: 100 mM sodium citrate 5.5; 20% PEG 3000 (w/v) (8IXT). For single-crystal microspectrophotometry of rTC-HOCbl/GSCbl, PACT conditions F5, identical to G5, but at pH 6.5, and F10, identical to F5, but sodium nitrate is substituted by 20 mM sodium potassium phosphate, were used.

For data collection, crystals were fished from the 96-well plate using nylon loops or lithoLoops (Molecular Dimensions and Mitegen, respectively) and frozen directly in the 100 K gaseous nitrogen stream at the tunable beam line BL26B1 at the SPring-8 facility (Hyogo) (43). Data were collected at wavelengths 1.7, 1.5, or 1.0 Å on an EIGER X 4M (DECTRIS). Diffraction images were processed using the XDS package (44). Crystals of rTC were of orthorhombic space group *P*2_1_2_1_2_1_. To obtain a structural model unbiased by molecular replacement-derived phases, it was decided to use the experimental phases from a single-wavelength anomalous dispersion dataset. The anomalous differences due to the presence of the Co atom in vitamin B_12_ were strong enough in the 1.2 Å resolution data collected at a λ = 1.0 Å to obtain a high-quality experimentally phased map using the SHELX package with hkl2map (45, 46). The model could be built by hand in COOT (https://www2.mrc-lmb.cam.ac.uk/personal/pemsley/coot/) (47) and refined using Phenix (https://phenix-online.org) (48, 49) to final R factors of 16.07 and 17.05 (R_work_/R_free_) (Table 1). Anomalous difference data collected at a wavelength of 1.7 Å could be used to locate the sulfur atoms in rTC including the sulfur atom of GSH located near the vitamin B_12_ molecule (Fig. 2). Figures were created using the PyMOL Molecular Graphics System, version 2.5 Schrödinger, LLC.

### Single-crystal microspectrophotometry and UV-visible spectroscopy

rTC was mixed with HOCbl or GSCbl and crystallized in PACT conditions F5 or F10, as mentioned above. Before X-ray data collection, the crystals were transferred to mother liquor supplemented with 20% glycerol, fished using a large 400 μm nylon loop, to minimize scattering of the loop, and frozen in the 100 K cryo stream mounted on an off-line single-crystal microspectrophotometry setup outside the BL26B1 beam line at the SPring-8 facility. The microspectrophotometer system consisted of a deuterium tungsten halogen light (Ocean Optics, DH-MINI), Cassegrain mirrors (Bunkoh-Keiki Co. Ltd), an optical fiber, and a linear charge-coupled device-array spectrometer (Ocean Optics, SD2000) (50). Absorption spectra were recorded at a wavelength of 178 to 879 nm and were baseline-corrected with a vitrified mother liquor blank and a dark reference.

Solution UV-visible absorption spectra were recorded at 293 K on a Shimadzu UV-1850 (Shimadzu) with a 1 cm 100 μl quartz cuvette. Equimolar amounts of 6 μM rTC and HOCbl were used and GSH was added at 1 mM. Time course UV-visible absorption spectra in solution were recorded at 293 K on a NanoDrop One (Thermo Fisher Scientific). Experiments were performed at concentrations of 200 μM, 200 μM, and 2 mM for rTC, HOCbl, and GSH, respectively. Samples were stored aerobically in the dark at 293 K. An aliquot was taken for spectroscopic analysis and discarded to prevent repeated cycles of light exposure and photodecomposition. Because the solution spectra and single-crystal microspectrophotometry spectra have different extinction coefficients and λ_max_ the spectra were normalized by surface area to be able to perform comparisons.

### MST affinity measurement

MST (51) experiments were performed on a blue/green Monolith NT.115 (NanoTemper Technologies) using NanoTemper premium capillaries. rTC was labeled with the blue *N*-Hydroxysuccinimide-coupled dye according to the manufacturer’s instructions (NanoTemper). The assay buffer was 100 mM Hepes pH 8.0; 100 mM NaCl; 0.05% Tween-20; 0.1% PEG 8000. Serial dilutions of 800 nM HOCbl and GSCbl were prepared in assay buffer. Fluorescently labeled rTC was added to the ligand serial dilutions at a final concentration of 625 pM. Experiments were performed according to the manufacturer’s instructions. Data were collected using the MO.Control software (https://nanotempertech.com) at high MST laser power and 100% excitation power. Traces were analyzed using the MO.Affinity Analysis v3.0.4 (https://nanotempertech.com) by calculating the fluorescence difference between the hot (red) and cold (blue) areas of the thermophoresis curves (Fig. 3*F*). Because of the limited sensitivity of the current setup, the concentration of labeled protein was above the *K*_D_; therefore, the *K*_D_ value that could be obtained is the lower limit of the *K*_D_ that could be determined under these experimental conditions (52).

### Sequence analysis

For the multiple sequence alignment in Fig. S1, the sequences of TC (rat, human, bovine, and mouse), gastric IF (rat, human and mouse), and HC (human and pig) were obtained from the UniProt database and aligned using Clustal Omega (53). The multiple sequence alignment was assembled with the experimentally obtained secondary structure using ESPript (54). The structure-guided sequence alignment of the α6/α7/α8 area in Fig. S6 was performed using Chimera (55). Sequence numbering is according to full-length rat TC (UniProt ID Q9R0D6).

## Data availability

Crystallographic data of the GSCbl- and HOCbl-bound rTC structures are deposited at the protein databank under PDB ID 8IXT and 8IXU, respectively.

## Supporting information

This article contains supporting information.

## Conflict of interests

The authors declare that they have no conflicts of interest with the contents of this article.
