# Supplementary material for: The structure of the rat vitamin B12 transporter TC and its complex with glutathionylcobalamin
Source: J Biol Chem. 2024 Apr 16;300(5):107289. doi: 10.1016/j.jbc.2024.107289 (PMC11107200; doi:10.1016/j.jbc.2024.107289)
Supplement: Figure S1 [file mmc1.pdf]

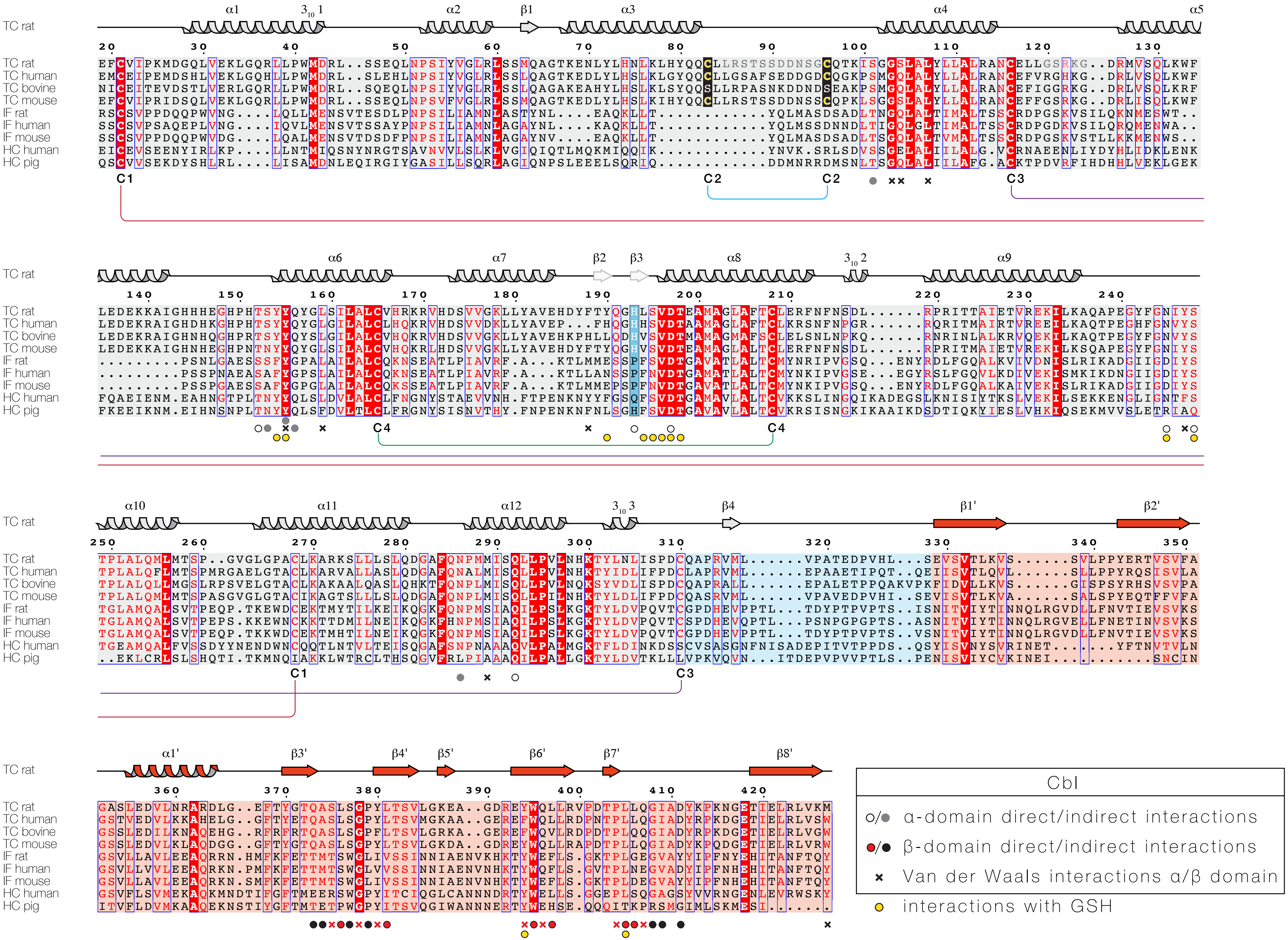

TC rat
α1 3₁₀1 α2 β1 α3 α4 α5
20 30 40 50 60 70 80 90 100 110 120 130
TC rat EFCVIPKMDGQLVEKLGQRLLPWMDRL..SSEQLNPSIYVGLRLSSMQAGTKENLYLHNLKLHYQQCLLRSTSSDDNSGCQTKISGGSLALYLLALRANCELLGSRKG..DRMVSQLKWF
TC human EMCEIPEMDSHLVEKLGQHLLPWMDRL..SLEHLNPSIYVGLRLSSLQAGTKEDLYLHSLKLGYQQCLLGSAFSEDDGDCQGKPSMGQLALYLLALRANCEFVRGHKG..DRLVSQLKWF
TC bovine NICEITEVDSTLVERLGQRLLPWMDRL..SQEQLNPSIYVGLRLSSLQAGAKEAHYLHSLKLSYQQSLLRPASNKDDNSEAKPSMGQLALYLLALRANCEFIGGRKG..DRLVSQLKRF
TC mouse EFCVIPRIDSQLVEKLGQRLLPWMDRL..SSEQLNPSVFVGLRLSSMQAGTKEDLYLHSLKIHYQQCLLRSTSSDDNSSCQPKLSGGSLALYLLALRANCEFFGSRKG..DRLISQLKWF
IF rat RSCSVPPDQQPWVNG....LQLLMENSVTESDLPNPSILIAMNLASTYNL....EAQKLLT............YQLMASDSADLTNGQLALTIMALTSSCRDPGSKVSILQKNMESWT..
IF human SSCSVPSAQEPLVNG....IQVLMENSVTSSAYPNPSILIAMNLAGAYNL....KAQKLLT............YQLMSSDNNDLTIGQLGLTIMALTSSCRDPGDKVSILQRQMENWA..
IF mouse SSCSVPPDQQPWVDG....LQALMENSVTDSDFPNPSILIAMNLAGAYNV....EAQKLLT............YQLMASDSADLTSGQLALTVMALTSSCRDPGSKVSTLLKKMENWS..
HC human EICEVSEENYIRLKP....LLNTMIQSNYNRGTSAVNVVLSLKLVGIQIQTLMQKMIQQIK............YNVK.SRLSDVSSGELALIILALG.VCRNAEENLIYDYHLIDKLENK
HC pig QSCVVSEKDYSHLRL....LISAMDNLEQIRGIYGASILLSQRLAGIQNPSLEEELSQRIQ............DDMNRRDMSNLTSGQLALIILAFG.ACKTPDVRFIHDHHLVEKLGEK
C1 C2 C2 C3
TC rat
α6 α7 β2 β3 α8 3₁₀2 α9
140 150 160 170 180 190 200 210 220 230 240
TC rat LEDEKKAIGHHHEGHPHTSYYQYGLSILALCVHRKRVHDSVVGKLLYAVEHDYFTYQGHLSVDTEAMAGLAFTCLERFNFNSDL......RPRITTAIETVREKILKAQAPEGYFGNIYS
TC human LEDEKRAIGHDHKGHPHTSYYQYGLGILALCLHQKRVHDSVVDKLLYAVEP...FHQGHHSVDTAAMAGLAFTCLKRSNFNPGR......RQRITMAIRTVREEILKAQTPEGHFGNVYS
TC bovine LEDEKKAIGHNHQGHPRTSYYQYSLGILALCVHQKRVHDSVVGKLLYAVEHKPHLLQDHVSVDTMAMAGMAFSCLELSNLNPKQ......RNRINLALKRVQEKILKAQTPEGYFGNVYS
TC mouse LEDEKKAIGHNHEGHPNTNYYQYGLSILALCVHQKRLHDSVVGKLLYAVEHDYFTYQGHVSVDTEAMAGLAFTCLERFNFNSDL......RPRITMAIETVREKILKSQAPEGYFGNIYS
IF rat .........PSNLGAESSSFYGPALAILALCQKNSEATLPIAVRF.A...KTLMMESSPFSVDTGAVATLALTCMYNRIPVGSQ...ENYRDLFGQALKVIVDNISLRIKADGIIGDIYS
IF human .........PSSPNAEASAFYGPSLAILALCQKNSEATLPIAVRF.A...KTLLANSSPFNVDTGAMATLALTCMYNKIPVGSE...EGYRSLFGQVLKDIVEKISMKIKDNGIIGDIYS
IF mouse .........PSSPGAESSAFYGPGLAILALCQKSSEATLPIAVRF.A...KTLMMEPSPFNVDTGAVATLALTCMYNKIPVGSQ...ENYRDLFGQALKAIVEKISLRIKADGIIGDIYS
HC human FQAEIENM.EAHNGTPLTNYYQLSLDVLALCLFNGNYSTAEVVNH.FTPENKNYYFGSQFSVDTGAMAVLALTCVKKSLINGQIKADEGSLKNISIYTKSLVEKILSEKKENGLIGNTFS
HC pig FKEEIKNM.EIHNSNPLTNYYQLSFDVLTLCLFRGNYSISNVTHY.FNPENKNFNLSGHFSVDTGAVAVLALTCVKRSISNGKIKAAIKDSDTIQKYIESLVHKIQSEKMVVSLETRIAQ
C4 C4
TC rat
α10 α11 α12 3₁₀3 β4 β1' β2'
250 260 270 280 290 300 310 320 330 340 350
TC rat TPLALQMLMTSP..GVGLGPACLKARKSLLLSLQDGAFQNPMMISQLLPVLNHKTYLNLISPDCQAPRVML......VPATEDPVHL...SEVSVTLKVS......SVLPPYERTVSVFA
TC human TPLALQFLMTSPMRGAELGTACLKARVALLASLQDGAFQNALMISQLLPVLNHKTYIDLIFPDCLAPRVML......EPAAETIPQT..QEIISVTLQVL......SLLPPYRQSISVLA
TC bovine TPLALQLLMGSLRPSVELGTACLKAKAALQASLQHKTFQNPLMISQLLPVLNQKSYVDLISPDCQAPRALL......EPALETPPQAKVPKFIDVLLKVS......GISPSYRHSVSVPA
TC mouse TPLALQMLMTSPASGVGLGTACIKAGTSLLLSLQDGAFQNPLMISQLLPILNHKTYLDLIFPDCQASRVML......VPAVEDPVHI..SEVISVTLKVA......SALSPYEQTFFVFA
IF rat TGLAMQALSVTPEQP.TKEWDCEKTMYTILKEIKQGKFQNPMSIAQILPSLKGKTYLDVPQVTCGPDHEVPPTL...TDYPTPVPTS..ISNITVIYTINNQLRGVDLLFNVTIEVSVKS
IF human TGLAMQALSVTPEPS.KKEWNCKKTTDMILNEIKQGKFHNPMSIAQILPSLKGKTYLDVPQVTCSPDHEVQPTL...PSNPGPGPTS..ASNITVIYTINNQLRGVELLFNETINVSVKS
IF mouse TGLAMQALSVTPEQP.TKKWDCEKTMHTILNEIKQGKFQNPMSIAQILPSLKGKTYLDVPQVTCGPDHEVPPTL...TDYPTPVPTS..VSNITVIYTINNQLRGVDLLFNVTIEVSVKS
HC human TGEAMQALFVSSDYYNENDWNCQQTLNTVLTEISQGAFSNPNAAAQVLPALMGKTFLDINKDSSCVSASGNFNISADEPITVTPPDS..QSYISVNYSVRINET.........YFTNVTVLN
HC pig ..EKLCRLSLSHQTI.TKMNQIAKKLWTRCLTHSQGVFRLPIAAAQILPALLGKTYLDVTKLLLVPKVQVN...ITDEPVPVVPTLS..PENISVIYCVKINEI.........SNCIN
C1 C3
TC rat
α1' β3' β4' β5' β6' β7' β8'
360 370 380 390 400 410 420
TC rat GASLEDVLNRARDLG..EFTYGTQASLSGPYLTSVLGKEA..GDREYWQLLRVPDTPLLQGIADYKPKNGETIELRLVKM
TC human GSTVEDVLKKAHELG..GFTYETQASLSGPYLTSVMGKAA..GEREFWQLLRDPNTPLLQGIADYRPKDGETIELRLVSW
TC bovine GSSLEDILNKAQEHG..RFRFRTQASLSGPFLTSVLGRKA..GEREFWQVLRDPDTPLQQGIADYRPKDGETIELRLVGW
TC mouse GSSLEDVLKLAQDGG..GFTYGTQASLSGPYLTSVLGKDA..GDREYWQLLRAPDTPLLQGIADYKPQDGETIELRLVRW
IF rat GSVLLAVLEEAQRRN.HMFKFETTMTSWGLIVSSINNIAENVKHKTYWEFLS.GKTPLGEGVAYYIPFNYEHITANFTQY
IF human GSVLLVVLEEAQRKN.PMFKFETTMTSWGLVVSSINNIAENVNHKTYWQFLS.GVTPLNEGVADYIPFNHEHITANFTQY
IF mouse GSVLLAVLEEAQRKN.SMFKFETTMTSWGLIVSSINNIAENVNHKTYWEFLS.GKTPLGEGVAYYIPFNHEHITANFTQY
HC human GSVFLSVMEKAQKMNDTIFGFTMEERSWGPYITCIQGLCANNNDRTYWELLS.GGEPLSQGAGSYVVRNGENLEVRWSKY
HC pig ITVFLDVMKAAQEKNSTIYGFTMTETPWGPYITSVQGIWANNNERTYWEHSE.QQQITKPRSMGIMLSKMESI......
Cbl
α-domain direct/indirect interactions
β-domain direct/indirect interactions
Van der Waals interactions α/β domain
interactions with GSH

Supplemental Figure S1 Multiple sequence alignment of transcobalamin (rat, human, cow and mouse), gastric intrinsic factor (rat, human and mouse) and haptocorrin (human and pig). Sequences were obtained from UniProt database entries Q9R0D6, P20062, Q9XSC9, O88968, P17267, P27352, P52787, P20061 and P17630, respectively. Sequence numbering is according to the sequence of full-length rat TC. Indicated above the sequence are the secondary structure elements of rTC and below are the disulfide bonds. Also indicated below are symbols indicating residues interacting with different ligands, the explanation can be found in the legend.
