# Supplementary material for: The structure of the rat vitamin B12 transporter TC and its complex with glutathionylcobalamin
Source: J Biol Chem. 2024 Apr 16;300(5):107289. doi: 10.1016/j.jbc.2024.107289 (PMC11107200; doi:10.1016/j.jbc.2024.107289)
Supplement: Figure S2 [file mmc2.pdf]

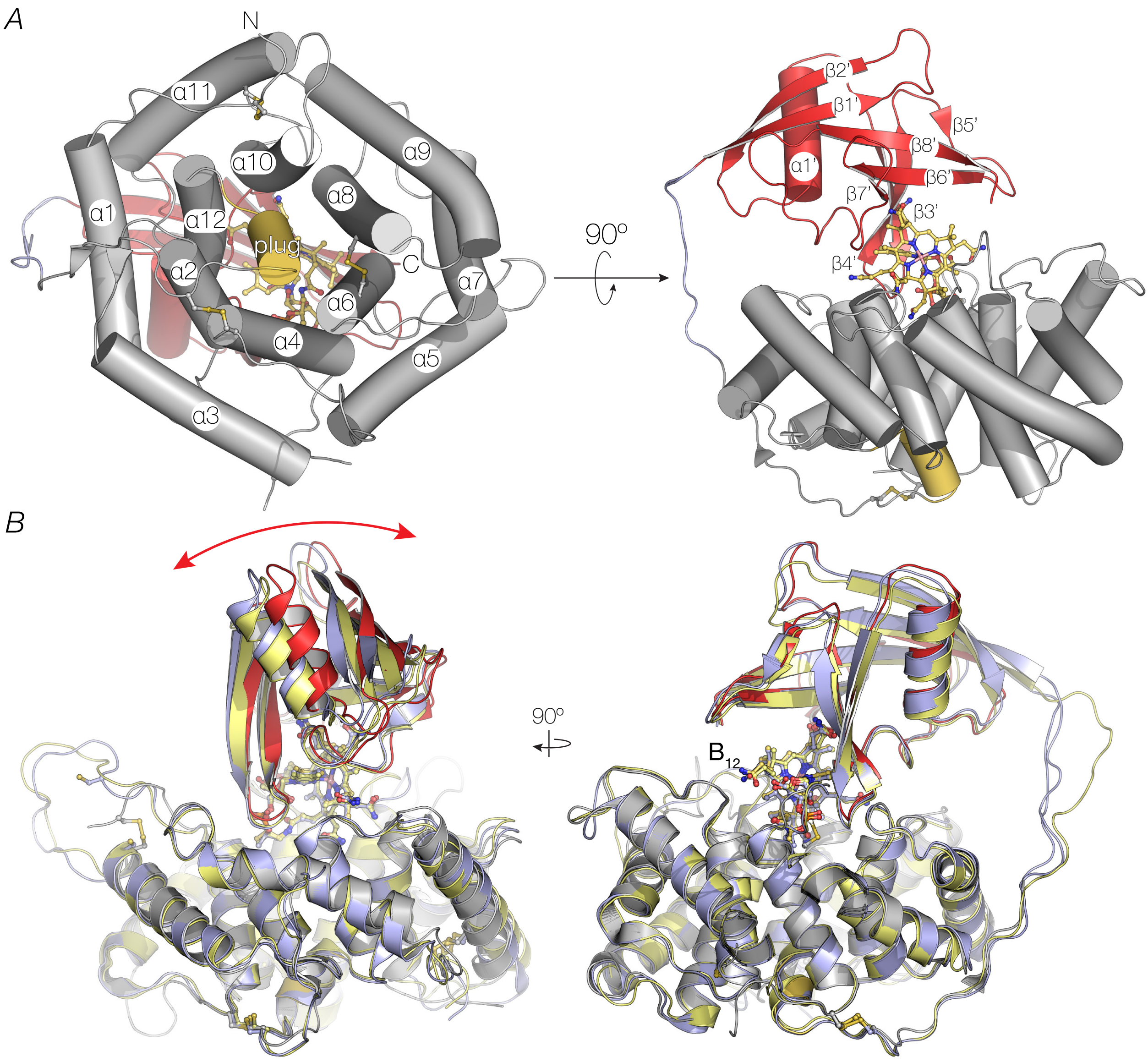

Supplemental Figure S2 Orthogonal views of TCs. (*A*) rTC as cartoons with helices as cylinders. In the N-terminal α domain (grey) the 12 α-helices (labeled by number) assemble into 6 antiparallel pairs that form a donut shape with a channel from the outside towards the B12 bound between α and β domain. The channel is blocked by the last α-helix of the α domain, which forms a plug. The plug continues into the interdomain linker (light blue) and then the C-terminal β domain (red). The β domain consists of two antiparallel β-strands, an α-helix followed by another pair of antiparallel β-strands. The disulfides are shown as ball-and-sticks. (*B*) Comparisons of different TCs with rTC colored as in *A*, bovine TC (2BBC) in yellow and human TC (2BB5) in blue.
