# Supplementary material for: The structure of the rat vitamin B12 transporter TC and its complex with glutathionylcobalamin
Source: J Biol Chem. 2024 Apr 16;300(5):107289. doi: 10.1016/j.jbc.2024.107289 (PMC11107200; doi:10.1016/j.jbc.2024.107289)
Supplement: Figure S3 [file mmc3.pdf]

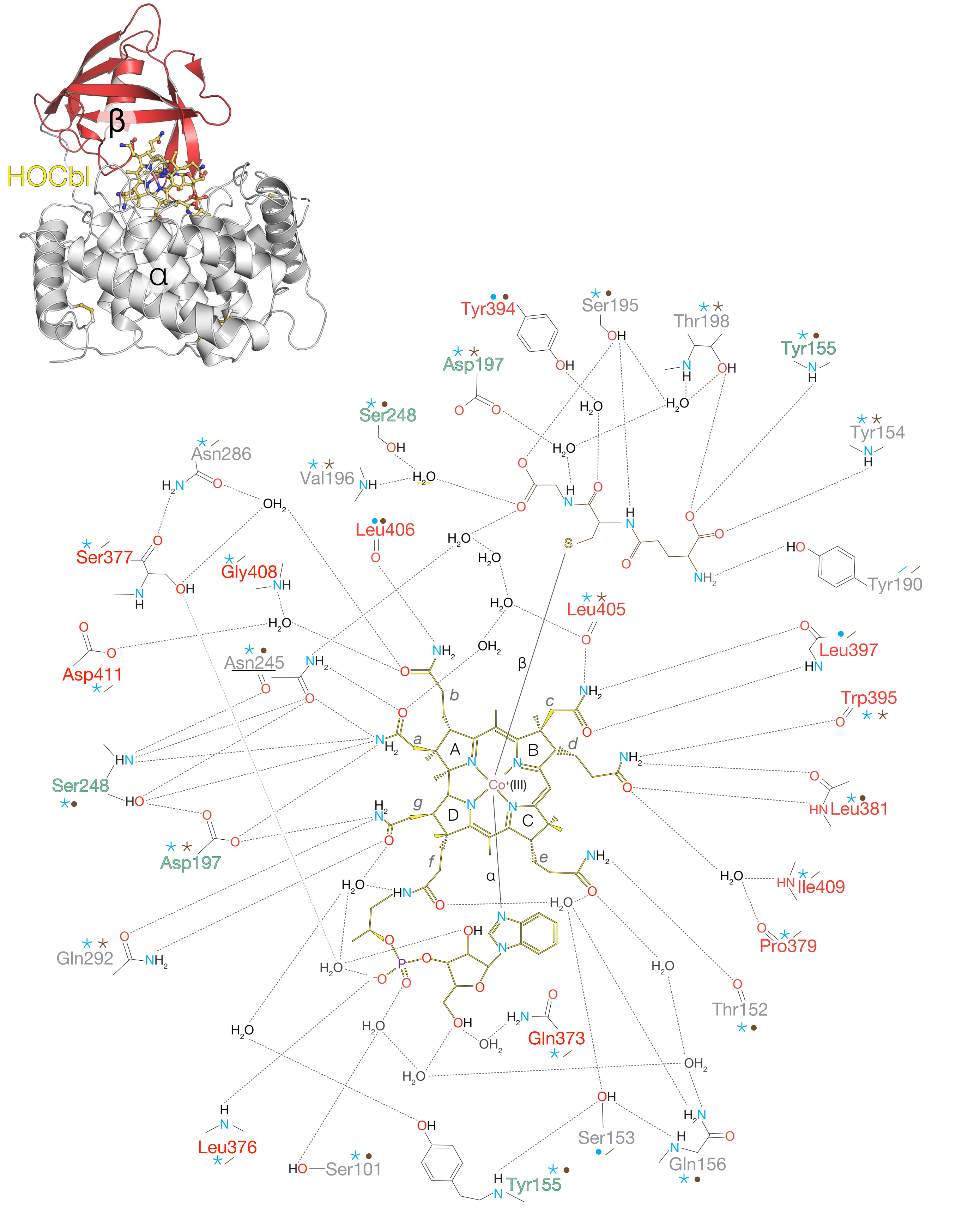

Supplemental Figure S3 Direct and solvent-mediated interactions between rTC and the Cbl-glutathione complex. Cbl, in the base-on conformation, is shown in yellow while glutathione is brown. Residues of the α domain are indicated in grey while the β-domain residues are red, green is used for α-domain residues shown twice for clarity if they interact with both Cbl and GSH. Sequence conservation among TC (rat, human, cow, mouse, orangutan, chimpanzee, dog, cat and pig) and TC/IF/HC (rat, human, cow, mouse/rat, human, mouse/human, and pig) are indicated at the residue label in blue and brown symbols, respectively. Symbols are as follows: strict sequence conservation (asterisk), sequence similarity (dot) and no sequence conservation (stroke). The inset in the top left shows the domain organization of rTC with α in white and β in red, HOCbl is indicated in yellow sticks.
