# Supplementary material for: The structure of the rat vitamin B12 transporter TC and its complex with glutathionylcobalamin
Source: J Biol Chem. 2024 Apr 16;300(5):107289. doi: 10.1016/j.jbc.2024.107289 (PMC11107200; doi:10.1016/j.jbc.2024.107289)
Supplement: Figure S4 [file mmc4.pdf]

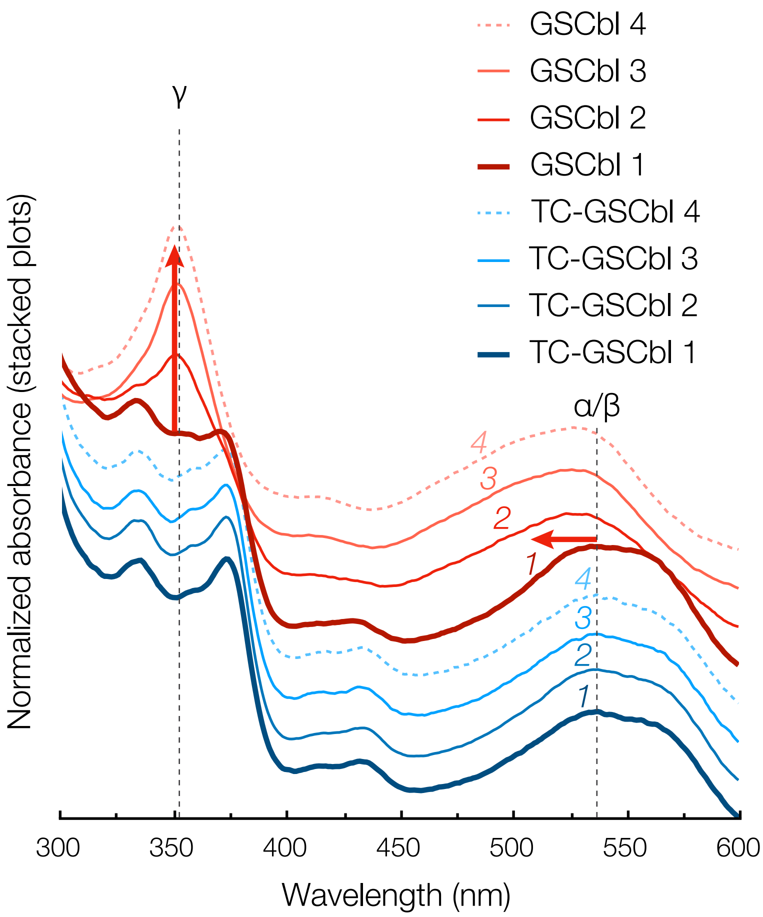

Supplemental Figure S4 Stacks (numbered) of normalized UV-Vis absorption spectra curves of free GSCbl (*shades of red*) and TC-bound GSCbl (*shades of blue*). Spectra of GSCbl were taken at time points 0, 72, 78 and 144 hours (1 to 4). Spectra for TC-GSCbl were taken at 0, 16, 90 and 162 hours (1 to 4). Concentrations were 200 μM, 200 μM and 2 mM for TC, Cbl and glutathione, respectively. Spectral changes in free GSCbl are indicated by red arrows. Incubation was performed under aerobic conditions in the dark.
