# Supplementary material for: The structure of the rat vitamin B12 transporter TC and its complex with glutathionylcobalamin
Source: J Biol Chem. 2024 Apr 16;300(5):107289. doi: 10.1016/j.jbc.2024.107289 (PMC11107200; doi:10.1016/j.jbc.2024.107289)
Supplement: Figure S5 [file mmc5.pdf]

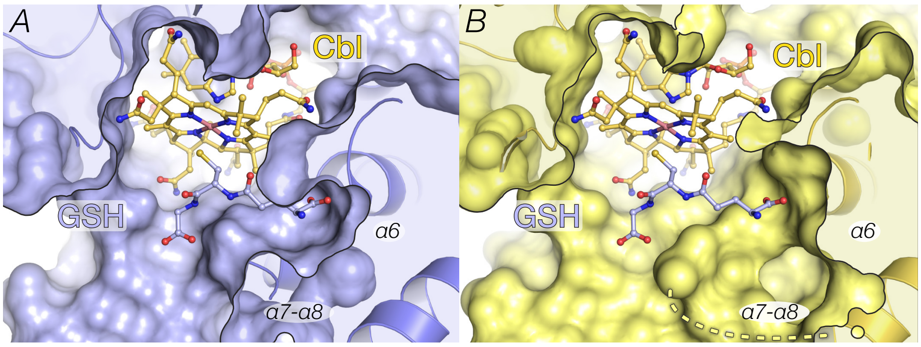

Supplemental Figure S5 Slice through of the substrate-binding pocket of human and bovine TC B_{12} transporters. (*A*) Human transcobalamin (7QBF) (38). (*B*) Bovine transcobalamin (2V3N) (26). B_{12} and GSH are indicated by yellow and blue sticks, respectively. Complexes are with cyanocobalamin where the histidine is displaced, opening the substrate-binding pocket. GSH is modeled by superimposing GSCbl onto the bound cobalamin.
