# Supplementary material for: The structure of the rat vitamin B12 transporter TC and its complex with glutathionylcobalamin
Source: J Biol Chem. 2024 Apr 16;300(5):107289. doi: 10.1016/j.jbc.2024.107289 (PMC11107200; doi:10.1016/j.jbc.2024.107289)
Supplement: Figure S6 [file mmc6.pdf]

A

B

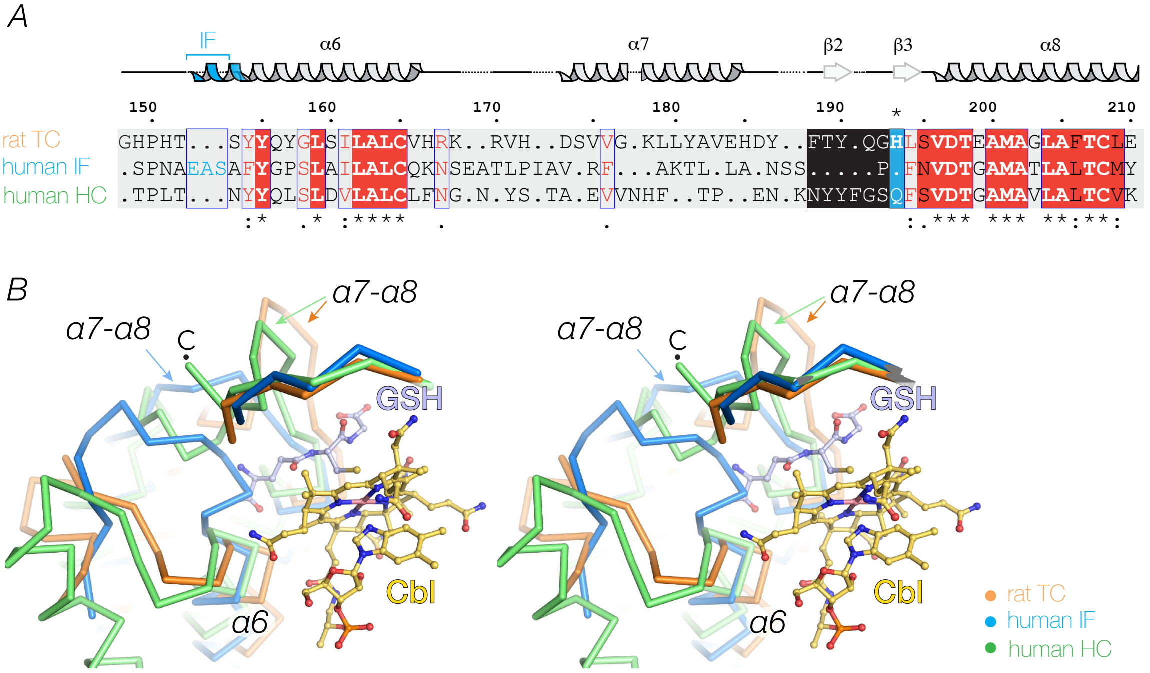

Supplemental Figure S6 Secondary structure elements surrounding GSCbl/Cbl. (*A*) Structure-guided multiple sequence alignment of rat TC and human IF and HC in the α 6-α8 area near the GSCbl/Cbl binding site. Numbering and secondary structure elements (*above*) according to rTC. Sequence conservation is indicated below. (*B*) Stereo image of the structure of the α6-α8 area in rTC, human IF and HC in orange, blue and green, respectively. The rest of the protein has been omitted for clarity. The protein is indicated as a Cα trace and B_{12} and GSH are show as yellow and blue sticks, respectively. Secondary structure elements are indicated and numbered according to Fig. S1. The C-terminus of the β-domain is indicated.
